# Transient oxidation of hazes a source of nutrients during the great oxidation event

**DOI:** 10.1038/s41598-025-13441-1

**Published:** 2025-08-11

**Authors:** L. Maratrat, A. Y. Jaziri, M. Millan, V. Moulay, L. Vettier, T. Govekar, A. Abello, N. Carrasco

**Affiliations:** 1 Université Paris-Saclay, UVSQ, CNRS, LATMOS, Guyancourt, 78280 France; 2 ENS-Paris-Saclay, Gif sur Yvette, 91190 France

**Keywords:** Atmospheric chemistry, Early solar system

## Abstract

The transition period of the Great Oxidation Event (GOE) saw the emergence of transient physical and chemical processes. In this context, photochemical organic hazes may have evolved in an atmosphere containing low concentrations of oxygen (< 10^− 5^/10^− 6^ Present Atmospheric Level). This coexistence of organic aerosols and oxygen traces induced an atmospheric reactivity which has so far been unexplored. Here, we show that the progressive rise of oxygen around the GOE triggered oxidation of organic aerosols suspected to be present at that time. We estimate that such transient mechanisms would have persisted over 60 to 100 Myrs during the GOE and possibly extended during the Archean. The main chemical markers of aerosol oxidation observed are urea, oxalic acid and carboxylic acid derivatives. These oxygen-bearing organics identified could have served as nutrients by existing ecosystems. This input of oxidized organic matter, estimated between 10^13^ and 10^15^ g of C.yr^− 1^ based on aerosol production yields, has probably been significant compared to oxygenic photosynthesis. Such mechanisms could also have diversified the pool of available nutrients, constituting an interesting vector of biodiversity for the primitive ecosystem.

**Supplementary Information:**

The online version contains supplementary material available at 10.1038/s41598-025-13441-1.

## Introduction

The Great Oxidation Event (GOE) is a major period estimated to have happened between 2.4 and 2.1 billion years ago and marked deep changes in the atmosphere of the Early-Earth. It went from a reduced atmosphere at the end of the Archean era to the significantly oxidized Proterozoic environment. In the Archean era, the atmospheric composition of the Early-Earth was notably reduced with very low O_2_ atmospheric level in comparison to present days. One of the best constraint on O_2_ proportion is given by S-MIF (Sulfur-Mass Independent Fractionation) observed in sulfide and sulfate minerals of Precambrian rocks^1,2^. Such isotopic anomalies are considered to result from SO_2_ photochemistry^3^ which is only active under a sufficiently reduced atmosphere^4^. Indeed, energetic UV light penetrates and dissociates SO_2_ only in a reduced atmosphere deprived of the ozone layer. An upper limit for oxygen is estimated in the Archean atmosphere between 10^− 6^ and 10^− 5^ PAL (Present Atmospheric Level) (0.2 ppm to 2 ppm)^5,6^. In this anoxic Archean world, the atmosphere is thought to be mainly dominated by N_2_ as well as significative amounts of CO_2_^7,8^ and CH_4_^6,8–10^ with no precise constraints on their respective abundancies. However, CH_4_ produced by methanogenesis was certainly more abundant in the early atmosphere than today^11,12^ in absence of photochemical oxidative sinks. More precisely, photochemical models estimate an order of magnitude of 1000 ppm for CH_4_ supposing an actual efficient microbial surface flux in the Archean era^13,14^.

This atmospheric composition with a significant amount of methane could have been favorable to the formation of organic hazes, considering the chemistry occurring in the atmosphere of Titan, the largest moon of Saturn^15^. Such hazes may have had a major influence on the global climate of the Early-Earth due to their radiative properties potentially leading to a global cooling or “anti-greenhouse” effect^16,17^ as well as a UV protection affecting NH_3_ photochemistry^18^. However, the existence of organic hazes is still an open question. S-MIF attenuation during the Mesoarchean^2,19–21^ and C^13^ depletion in sediments^11,12,22^ support episodic haze formations. With a very different approach, laboratory experiments reproducing the Archean photochemistry^23,24^also argue in favor of the existence of organic hazes during this era. Then, the GOE (2.4–2.1 Ga) has been detrimental to anoxygenic methanogens triggering a decrease in methane surface emission, thus progressively leading to the disappearance of organic hazes.

During this transition, transient geo-chemical processes influenced the Early-Earth system on a global scale, playing an important role in its evolution. Our work focuses on the peculiar evolution of photochemical hazes. In this transition regime, notably in the beginning of the GOE, organic hazes coexisted with traces of oxidizing species due to the rise of oxygen produced by photosynthetic life. These interactions with trace atmospheric oxidizers induced chemical transformations on the aerosols which has never been addressed so far. More precisely, the atmosphere could have offered favorable conditions for the occurence of haze heterogenous oxidation processes. The concomitant presence of photochemical aerosols produced efficiently by methane photochemistry on the one side and a more important concentration of oxidized species at the surface on the other side could have triggered efficient oxidation ageing processes as those observed in the present Earth atmosphere^25^. Yet, such atmospheric heterogenous mechanisms constitute an interesting source of oxygen and nitrogen-bearing organic molecules^26^ before any interaction with liquid water, providing a global input of evolved organic matter at the surface potentially utilizable by the biosphere.

To investigate experimentally these interactions and their implications in the context of the Archean and the GOE, we considered a starting pool of reduced haze analogues containing nitrogen (haze particles made of only C_x_H_Y_N_z_ molecules) as a model case of reducing matter for Early-Earth hazes. We exposed this material to a weakly oxidative N_2_ atmosphere plausible for the Archean (and the beginning of the GOE) in term of oxidation level (1 ppm of H_2_O and 0.1 ppm of O_2_) to study its evolution in such atmospheric conditions. Then, we searched for the presence of oxygen-bearing molecules, which, in this case, are directly indicative of an oxidation process. The analytical characterization of these organic molecules has been performed using gas chromatography coupled with mass spectrometry (GC-MS).

## Results

### Identification of oxygen-bearing molecules: a tracer of aerosol reactivity in weakly oxidative conditions

Eight oxygen-bearing compounds are revealed in our samples by analyzing the GC-MS data.

**Fig. 1:**
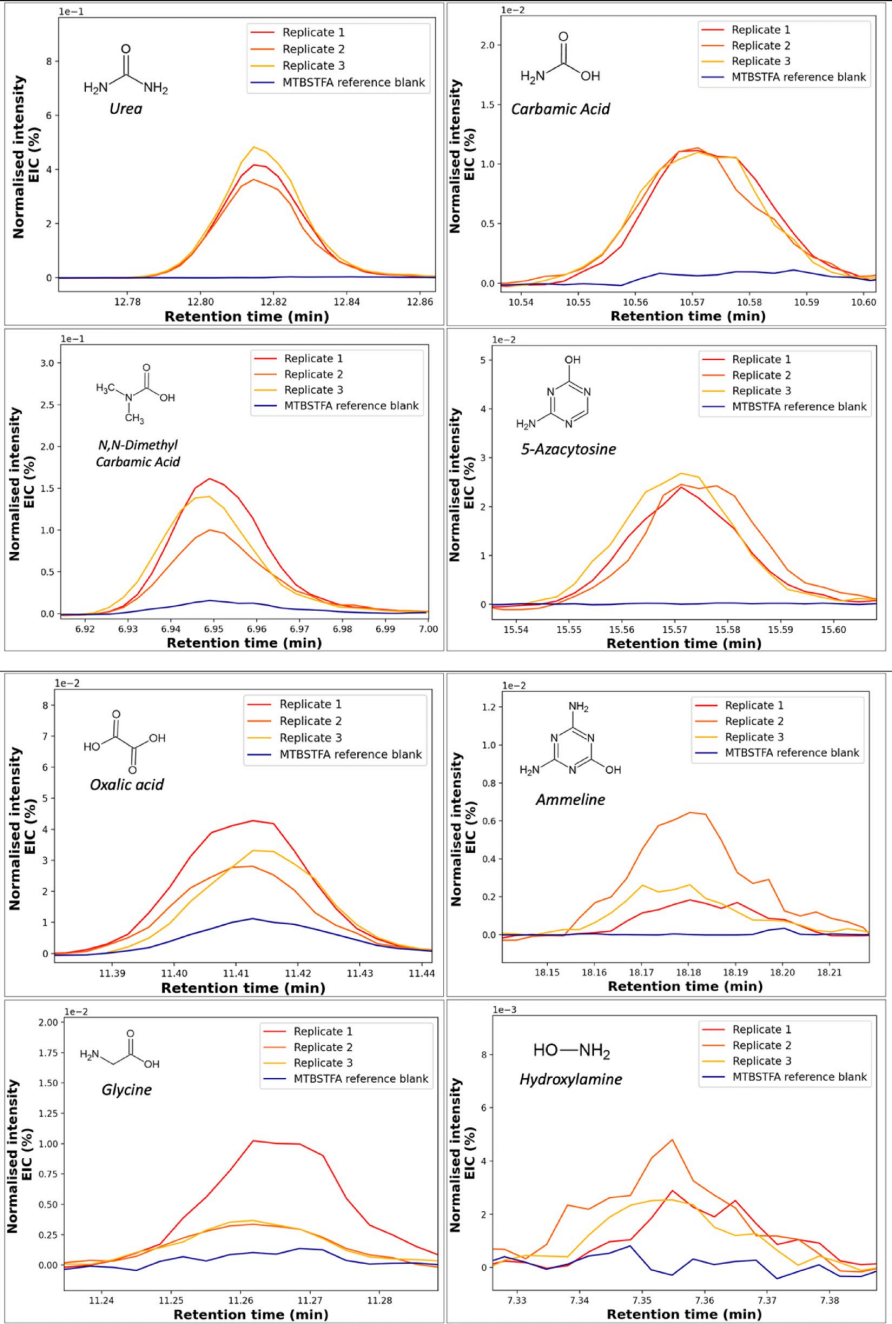
Extracted Ion Chromatograms (EIC) for the different oxygenated species identified. The fragments used for the sub-figures are respectively: m/z = 231 for urea, m/z = 232 for carbamic acid, m/z = 146 for N, N-Dimethyl carbamic acid, m/z = 283 for 5-Azacytosine, m/z = 261 for oxalic acid, m/z = 298 for ammeline, m/z = 246 for glycine, and m/z = 132 for hydroxylamine. The signals have been normalized by the maximum intensity of the internal standard peak (Naphtalene-D8) to correct variations in the response of the instrument. For clarity, the signals have also been synchronized (to fix the maximum of the peaks at the same retention time) and their baseline have been corrected.

The measurements were repeated in triplicates and blanks were performed to confirm that the molecules of interest were distinct from any contamination sources that may have occurred during the storage and the sample preparation protocol prior to the injection into the GC. The sub-figures presented in Fig. 1 show the comparative chromatograms between the blank and the haze analogs after storage under the chosen experimental conditions (0.1 ppm O_2_, 1ppm H_2_O). The Extracted Ion Chromatograms (EIC) related to each oxygen-bearing compound show a significant increase of their relative intensities compared to the blank. The m/z chosen for these EIC are specified (Fig. 1). For all the molecules, the signal over noise ratio is superior to 3 which is meaningfull.

The eight species targeted were identified through their mass spectra (see Figure S-1 in supplementary material) compared to their corresponding reference from the National Institute of Standard and Technology (NIST) spectral database. The identification of the molecules were also confirmed using the retention times of the standard molecules ran within the same GC-MS conditions. To strictly confirm these identifications and the oxidation process involved, we voluntary exposed the sample to the ambient atmosphere. This increased the efficiency of the oxidation processes and thus subsequently increased the abundance of the oxygenated molecules produced. In such conditions, the mass spectra obtained for the oxidized sample matched the NIST standard (see supplementary materials, Figure S-2).

**Fig. 2:**
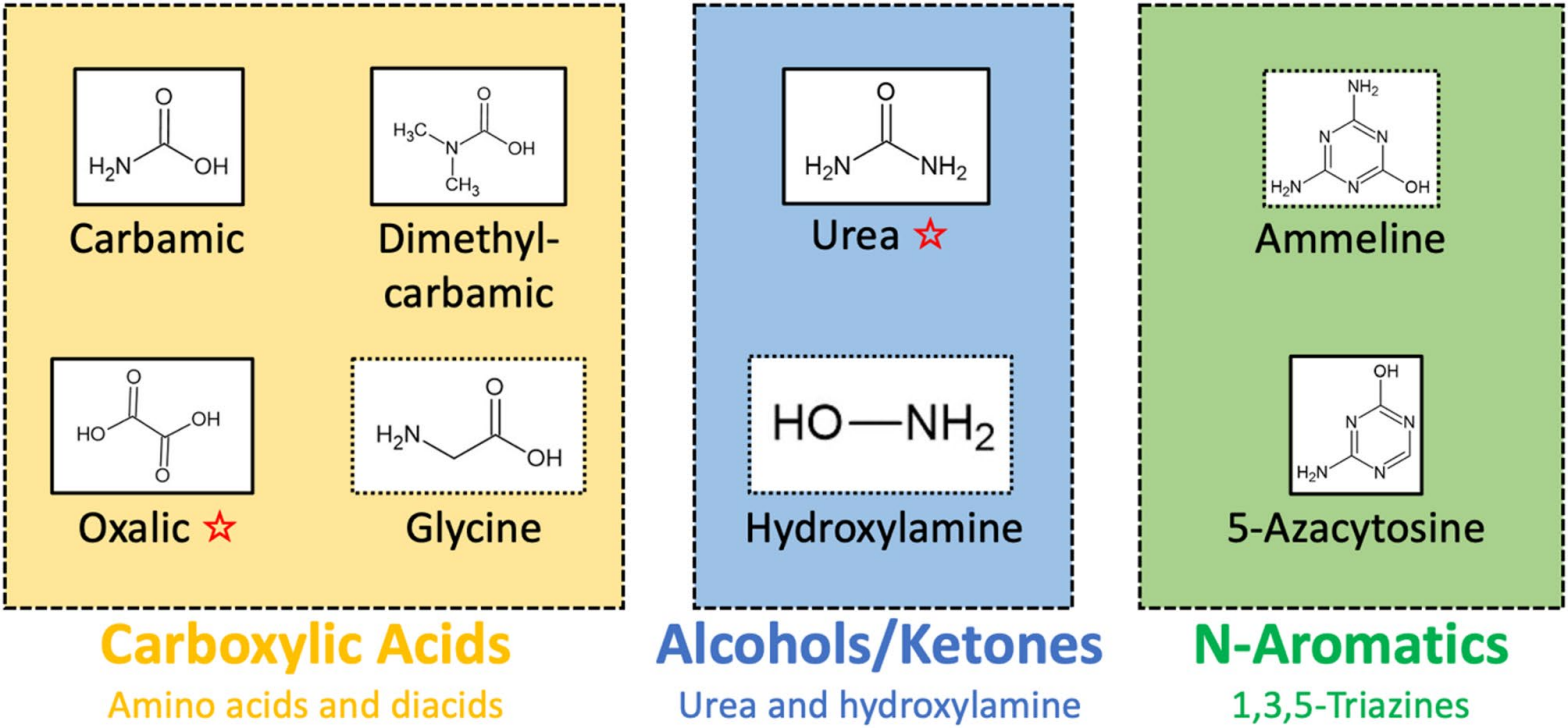
Chemical structure of the eight oxygen-bearing molecules identified in this study, classified by chemical functions and/or structure similarities. The red star designates the molecules with the most intense signal.

Figure 1 revealed the formation of eight oxygenation products: carbamic acid, dimethylcarbamic acid, oxalic acid, glycine, urea, hydroxylamine, ammeline and 5-azacytosine. Such chemical structures are represented in Fig. 2. The products observed mainly result from the reactivity of OH radicals on the organic haze matrix. OH radicals are known to react on unsaturation to produce keton derivatives^27^ as urea. Moreover, OH radicals also add on aromatic hydrocarbons leading to the formation of hydroxy aromatics^28^ like ammeline and 5-Azacytosine. These remarks highlight the common points existing between the reactivity of the organic haze matrix studied and the one of reduced Volatile Organic Compounds in the Earth troposphere mainly controlled by OH radicals^25^.

Figure 2 shows the wide diversity of the oxygen-bearing organic functions formed through oxidation of the reduced haze analogue with the presence of carboxylic acids, ketone, and alcohols. These findings are consistent with previous works that studied the properties and the reactivity of reduced haze analogues after hydrolysis in acidic or basic conditions, and have shown the formation of amino acids, diacids and urea^29–31^. Results presented in this study show that atmospheric dry mechanisms lead to the same species, without the assistance of liquid water. In addition, we show that 1,3,5-triazines are the only oxygen and nitrogen-bearing aromatic species identified here. Their formation could be favored by the high abondance of Triazine in the aerosol analogues as suggested by several studies^32–35^.

These results and the formation of oxygen species confirm the possibility for reduced organic compounds contained within an aerosol matrix to react with traces atmospheric oxidant species up to the ppm level. The quantity of oxidized products forms has not been specifically quantified here. On exposure times longer than the ten days performed in this study more important yields could be reached. After several months, which represent the typical degradation timescale of haze organics in presence of humidity^36^oxidized molecules, and more particularly urea which is the most abundant one, are present in significative proportions in the bulk material. A previous study notably estimated an order of magnitude of 1 mg/g for the mass fraction of urea in similar organic hazes after exposure to ambient air^37^.

Our results and the previous remarks thus provide experimental evidences of the important role played by atmospheric oxidation processes in the Archean and at the beginning of the GOE.

### Aerosol oxidation during the GOE: existence and timescales

Here we investigate the existence of aerosol oxidation during the GOE by constraining more precisely in time the interval during which such processes could have persisted. To do so, we define two necessary conditions for aerosol oxidation, which are the existence of organic aerosols and a sufficient proportion of oxygen reactant in the atmosphere.

Based on our experimental results, we take an order of magnitude of 0.1 ppm as a limit in O_2_ content to trigger oxidation on aerosols.

For organic aerosols, we focus our approach on the variation of atmospheric composition and its associated uncertainty. One key parameter for haze production is the ratio CH_4_/CO_2_. Based on a previous experimental study, a criterion for aerosol formation is roughly chosen at CH_4_/CO_2_ > 0.1^23^. While this criterion works for a majority of mixtures, it can sometimes be restrictive and several counter-examples can be cited: for instance H_2_-CO_2_-N_2_ mixtures without CH_4_ are also known to produce organic films^24^. Nevertheless, we used this criterion as an underestimation of the existence of aerosols during the GOE and the Archean.

**Fig. 3:**
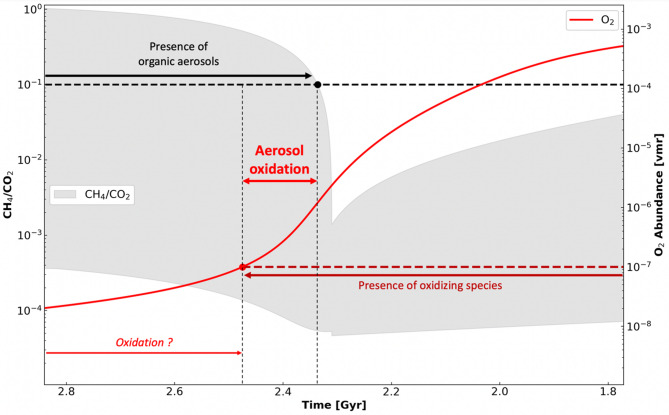
On the left axis is shown the uncertainties of the ratio CH_4_/CO_2_ with times. The black dashed line indicates the condition used for aerosol existence CH_4_/CO_2_ > 0.1. On the right axis the O_2_ volume mixing ratio (vmr) is plotted in red. The red dashed line reminds the O_2_ exposure value for the aerosols performed in this work and the criteria used for oxidation. The time line here is voluntary restricted between 1.8 and 2.8 Gyrs to focus on the GOE. In the Early Archean before 2.8Gyrs, the O_2_ vmr and the CH_4_/CO_2_ do not change significatively.

Figure 3 shows the variations of O_2_ volume mixing ratio (vmr) as well as the uncertainties on the ratio CH_4_/CO_2_ during the Archean and the GOE. O_2_ vmr is calculated using the approach described in previous modelling studies^38^. It takes into account phenomena such as surfaces flux from methanogenesis and photosynthesis, loss by hydrogen escape, and photochemical processes. Moreover, temporal uncertainties on CO_2_ and CH_4_ have been taken from the following review^4^. Such uncertainties allow to estimate the upper ($\:{\sigma\:}_{+})$ and lower ($\:{\sigma\:}_{-})$ limits for the ratio CH_4_/CO_2_ thanks to the following relations $\:{\sigma\:}_{+}\left(t\right)=\frac{\text{max}{CH}_{4}\left(t\right)}{\text{min}{CO}_{2}\left(t\right)}$ and $\:{\sigma\:}_{-}\left(t\right)=\frac{\text{min}{CH}_{4}\left(t\right)}{\text{max}{CO}_{2}\left(t\right)}$.

The envelop on the ratio CH_4_/CO_2_ shown in Fig. 3, suggests a likely formation of organic aerosols during the Archean and the GOE. The difference between the threshold for aerosol formation CH_4_/CO_2_ = 0.1 (black dashed line) and the upper limit value of this same ratio is quite significative before the GOE. This difference starts decreasing progressively in the GOE but remains in favor of aerosol formation until the 0.1 threshold is crossed. The intersection between the upper envelope of the CH_4_/CO_2_ ratio and the 0.1 threshold gives a temporal limit for aerosol existence in the GOE (black dot Fig. 3). This critical point is reached at 2.34 Gyrs, so approximatively 0.06 Gyrs after the beginning of the GOE (2.4 Gyrs). Such duration is not negligible compared with the 0.3 Gyrs of the GOE in total and represents 20% of this value. Beyond 2.34 Gyrs, the atmosphere is too oxidated and methane depleted to trigger organic aerosol formation.

Furthermore, the temporal variation of O_2_ volume mixing ratio (vmr) in Fig. 3 reveals a sufficient level of atmospheric oxidizers to trigger oxidation processes during the existence period of aerosols. The oxygen threshold value of 0.1 ppm$\:\:\approx\:{10}^{-7}$vmr for oxidation is reached around 2.47 Gyrs, and ensures favorable conditions for aerosol oxidation between 2.47 and 2.34 Gyrs. These results suggest that aerosol oxidation processes could have persisted during the GOE over significative timescales of 100 Myrs. Figure 3 also argues for a possible extension of the aerosol oxidation window in the Archean, before the GOE. The Archean “steady-state” value of oxygen is indeed around $\:{10}^{-8}$ vmr $\:\approx\:\:$0.01ppm and quite close to the oxidation threshold of 0.1 ppm. This concentration is not incompatible with oxidation, notably if we assume longer aerosol exposure times (taking into account their sedimentation and residence times at the surface) than the ten days performed in this work.

To go further, the prediction of O_2_ concentrations strongly relies on the net production of O_2_ by photosynthesis of the Archean marine ecosystem $\:N$ (see methods). Figure 3 corresponds to a favorable case for oxidation assuming an already efficient $\:N$ value, ten times inferior to the net productivity of the modern marine ecosystem $\:\zeta\:$. However, with a less efficient ecosystem i.e. a smaller $\:N$, the Archean steady-state O_2_ value decreases and the oxidation window is shortened due to a faster GOE tranisition (see supplementary materials). In this model the most unfavorable case is $\:N={10}^{-3}\zeta\:$ which corresponds to the minimum value of $\:N$ leading to the appearance of the GOE in the simulation and therefore to coherent solutions. This critical value of $\:N$ gives an estimation of the shortest possible oxidation window with this approach. In this latter case, the oxidation occurs between 2.34 and 2.28 Gyrs and lasts 60 Myrs.

To conclude on the aerosol oxidation timescale, in all the scenarios described above, aerosol oxidation processes could have persisted during significant timescales between 60 and 100 Myrs. Depending on the net primary productivity by oxygenic photosynthesis, $\:N$, the oxidation window could have been more precisely in the ranges 2.47–2.34 or 2.34–2.28 Gyrs with a potential extension in the Archean for the former case. The typical timescales highlighted here are therefore longer enough to envisage global impacts for the Early-Earth and its ecosystem.

## Discussion

### Impact of oxidation processes on early-earth ecosystems

The atmospheric oxidation of the organic haze, as previously shown in this work, would benefit the biosphere by providing directly or indirectly nutrients. Here we want to give examples of metabolisms susceptible to take advantage of these oxidized organic resources and consider the specific role of the main molecules observed.

The actual knowledge of the Neoarchean ecosystem is limited. However geological evidences as well as environmental constraints on available resources have further improved our representation of this system. Isotopic anomalies, recorded in Archean rocks on carbon^12^ sulfur^39^ and iron^40,41^have been attributed to biological fractionation caused by methanogens, sulfur, and iron-metabolising microorganisms, respectively. The discovery of cyanobacteria microfossils^42^ coupled with phylogenetic approach^43^ also suggests the existence of oxygenic photosynthesis since the Paleoarchean.

In this already diversified Archean ecosystem as we currently understand it, several heterotroph organisms are known to use oxidized organic matter. For instance, Methanogens reduce carbon to produce methane and consequently need an input of a higher oxidation state carbon. If CO_2_ is a common oxidized resource for methanogens, other carbon substrates such as methyl organics, acetate, or formate^43,44^ are also used in the methanogenesis. Sulfur-reducing^45^ and iron-reducing bacteria^46^ are other examples of anaerobic organisms working with oxidized organic matter. In these metabolisms, sulfur and iron are further reduced by the oxidation of carbon into CO_2_. For both sulfur and iron bacteria types, oxidized organic substrates can take many diversified forms^47,48^. These non-exhaustive examples highlight the importance of oxidized organics in general and carboxylic acid derivatives in particular. This intermediary redox state indeed allows to be implied in very different metabolisms performing carbon oxidation as well as carbon reduction. The detection of carboxylic acids in our experiment confirms the possibility for aerosol oxidation mechanisms to be a global source of relevant organic nutrients in addition to photosynthesis.

Moreover, among the molecules previously identified, oxalic acid and urea have a distinct influence on the biosphere due to their inherent chemical properties. A significative indirect influence of oxalate for Early-Earth ecosystems is its ability to solubilize the iron(II) contained in rocks thanks to the formation of a divalent complex^49^. This strong affinity of oxalate for iron could have increased the availability of Fe^2+^ nutrients for iron oxidizing phototrophs present in the Early-Earth oceans. Urea on the other side could have been a privileged nutrient for cyanobacteria. Modern cyanobacteria easily fix nitrogen resources and more particularly urea thanks to urease enzyme which hydrolyses urea into ammonia and carbon dioxide^50^. Urease-encoding genes are found in the cyanobacteria genome^51^but the question of nitrogen fixation in early-lineage of evolution remains an issue to address^52^. Finally, another important indirect impact of such oxidized organic matter relies on its reactivity notably on mineral surfaces. Evaporation/precipitation cycles on mineral induces a particularly efficient chemistry susceptible to transform the previous molecules into a second generation of organics having a different impact^53^. The question of evolution of organic matter at the surface is therefore central to better assess its potential impacts at planetary scale.

In conclusion, all these elements point out the nutritive role of oxidized organic matter induced by aerosol oxidation processes for early ecosystems. Such oxygenated molecules constitute a new source of carbon for the heterotroph species whose contribution should be considered in the global carbon cycle. This input of oxidized organic matter from aerosols could have been significant for the heterotroph organisms i.e. comparable to the flux of C produced by primary oxygenic photosynthesis. Experiments^23^ and models^54^ estimate a flux of organic matter from haze at the surface of the Early-Earth between 10^13^ and 10^15^ g of C.yr^− 1^, which corresponds respectively to 1/1000 and 1/10 of primary carbon production by modern marine ecosystem. Such values are within the order of magnitude of the primary production of the Early-Earth ecosystem which is described in the literature as less efficient^55^ compared to nowadays. Moreover, such mechanisms could have provided different types of organic substrates than photosynthesis thus generating a diversified pool of available nutrients for the ecosystem. This wide diversity of molecules from different sources and processes have probably been an important vector of biodiversity for the primitive ecosystem. The understanding of the occurrence and the dynamic of the GOE also lies in a more detailed description of the Archaean ecosystem and its biodiversity.

## Methods

### Aerosol synthesis

Tholins were produced from a N_2_/CH_4_ mixture (96.5:4.5) in an AC cold Plasma setup called PAMPRE^56^ at a pressure of 1mbar, a flowrate of 55 sccm (Standard Cubic Centimeter per Minutes) and a power of 30 W. Such experiment aims to simulate the upper layer of Early-Earth atmosphere, in which the incoming rays are sufficiently energetic to trigger a complex chemistry leading to the formation of solid particles.

Then the aerosols were collected and stored for ten days under a controlled atmosphere (0.1 ppm O_2_ and 1ppm H_2_O) in a glovebox (Jacomex BS 531). This proportion in O_2_ represents an experimental limit that cannot be exceeded due to the capacity of the glovebox and the residual vacuum in the plasma chamber which is under primary pumping (10^− 3^ mbar). Such exposure aims to study chemical evolution of reduced aerosols in the atmosphere and/or at the surface after their sedimentation. Oxidizing species such as OH radicals are indeed susceptible to react with reduced organic hazes.

### Analytical protocol

Prior to injection into the GC-MS, the collected hazes were derivatized with a solution of MTBSTFA: DMF (acronyms for DiMethylFormamide and *N*-*Tert*-ButyldiméthylSilyl-*N*-méthylTriFluoroAcétamide) (4:1 in volume) containing naphtalene-d8 as an internal standard. This derivatization protocol was used here to enhance the detection of the polar oxidized molecules by replacing their labile hydrogens into a *Tert*-ButylDimethylSilyl group (*t*-BDMS), making them more amenable to GC-MS. The reaction was performed at 75 °C during 15 min with a concentration of 3 mg of sample for 40$\:\:\mu\:L$. Three replicates of this derivatized solution were prepared as well as a blank reference sample composed of an empty vial stored in the same conditions and duration as the aerosols. A derivatization was also performed in this vial and constitute the blank reference sample plotted in Fig. 1. The DMF (Purity > 99%) and MTBSTFA reagents (Purity > 99%) as well as the naphtalene-d8 standard (99%D, Purity > 98%) were purchased at sigma-Aldrich.

Finally, 1 $\:\mu\:L$ of the derivatized solution was injected in the GC-MS for analysis (Thermo scientific Trace 1300 GC coupled with a Thermo scientific ISQ Ion Single Quadrupole mass spectrometer) on a RT-X20 capillary column. The temperature of the GC column was initially 60 °C followed by two ramps of 5 °C.min^− 1^ up to 150 °C and 10 °C.min^− 1^ up to 300 °C, and finally held at this temperature for 5 min. The split of Helium gas (Air liquide, purity > 99.999%) was 20 ml.min^− 1^. The mass spectrometry measurements were performed using an electronic impact ionization of 70 eV over a range of 40–600 mass units. The temperature of the line transfer and the source were both set at 300 °C.

The retention time validation of the different compounds has been performed on the same instrument using the same analytical method. The chemical standards urea, glycine, oxalic acid, ameline, 5-azacytosine and hydroxylamine used for this measurements were also purchased at Sigma-Aldrich, except ameline (Fischer Scientific).

### O_2_ variation model

The temporal evolution of O_2_ vmr is calculated using the following system of coupled differential equations involving the functions $\:M\left(t\right)$ and $\:O\left(t\right)$ which represent respectively the absolute quantities of CH_4_ and O_2_ in mol. $\:{{\Omega\:}}_{{O}_{2}},\:r$, and $\:{{\Psi\:}}_{{O}_{2}}$are functions of $\:O\left(t\right)$, their parametrization is described more precisely in the following study^38^. $\:s=2.03\times\:{10}^{-5}$yr^−1^ is a fixed parameter for the hydrogen loss, whereas $\:N\:$represent the annual production of oxygen by marine photosynthesis. For the simulation Fig. 3, we assume a marine photosynthesis productivity $\:N=3.5\times\:{10}^{14}\:$mol O_2_.yr^−1^ ten times inferior to modern marine ecosystem.

$$\:\left\{\begin{array}{c}\frac{dM}{dt}=\frac{1}{2}{{\Omega\:}}_{{O}_{2}}\left(N+r\right)-sM-\frac{1}{2}{{\Psi\:}}_{{O}_{2}}{M}^{0.7}\:\:\left(1\right)\\\:\:\:\:\:\:\:\:\frac{dO}{dt}={\text{N}{{\Omega\:}}_{{O}_{2}}-(1-{\Omega\:}}_{{O}_{2}})r-sM-{{\Psi\:}}_{{O}_{2}}{M}^{0.7}\:\:(2)\end{array}\right.$$

## Supplementary Information

Below is the link to the electronic supplementary material.

Supplementary Material 1

## Data Availability

GC-MS data used and/or analysed during the current study available from the corresponding author on reasonable request.

## References

[1] Farquhar, J., Bao, H. & Thiemens, M. Atmospheric influence of earth’s earliest sulfur cycle. *Science* **289**, 756–758 (2000). 10926533 10.1126/science.289.5480.756

[2] Farquhar, J. et al. Isotopic evidence for mesoarchaean anoxia and changing atmospheric sulphur chemistry. *Nature* **449**, 706–709 (2007). 17928857 10.1038/nature06202

[3] Farquhar, J., Savarino, J., Airieau, S. & Thiemens, M. H. Observation of wavelength-sensitive mass-independent sulfur isotope effects during SO2 photolysis: implications for the early atmosphere. *J. Geophys. Res. Planet*. **106**, 32829–32839 (2001).

[4] Catling, D. C. & Zahnle, K. J. The archean atmosphere. *Sci. Adv.* **6**, eaax1420 (2020). 32133393 10.1126/sciadv.aax1420 PMC7043912

[5] Pavlov, A. A. & Kasting, J. F. Mass-independent fractionation of sulfur isotopes in archean sediments: strong evidence for an anoxic archean atmosphere. *Astrobiology* **2**, 27–41 (2002). 12449853 10.1089/153110702753621321

[6] Zahnle, K. J., Claire, M. W. & Catling, D. C. The loss of mass-independent fractionation in sulfur due to a paleoproterozoic collapse of atmospheric methane. *Geobiology* **4**, 271–283 (2006).

[7] Sheldon, N. Precambrian paleosols and atmospheric CO2 levels. *Precambrian Res.* **147**, 148–155 (2006).

[8] Driese, S. G. et al. Neoarchean paleoweathering of tonalite and metabasalt: implications for reconstructions of 2.69Ga early terrestrial ecosystems and paleoatmospheric chemistry. *Precambrian Res.* **189**, 1–17 (2011).

[9] Zahnle, K. J., Gacesa, M. & Catling, D. C. Strange messenger: A new history of hydrogen on earth, as told by Xenon. *Geochim. Cosmochim. Acta*. **244**, 56–85 (2019).

[10] Kurokawa, H., Foriel, J., Laneuville, M., Houser, C. & Usui, T. Subduction and atmospheric escape of earth’s seawater constrained by hydrogen isotopes. *Earth Planet. Sci. Lett.* **497**, 149–160 (2018).

[11] Stueeken, E. E. & Buick, R. Environmental control on microbial diversification and methane production in the mesoarchean. 10.1016/j.precamres.2017.11.003 (2018).

[12] Ueno, Y., Yamada, K., Yoshida, N., Maruyama, S. & Isozaki, Y. Evidence from fluid inclusions for microbial methanogenesis in the early Archaean era. *Nature* **440**, 516–519 (2006). 16554816 10.1038/nature04584

[13] KHARECHA, P., Kasting, J. & Siefert, J. A coupled atmosphere–ecosystem model of the early archean Earth. *Geobiology* **3**, 53–76 (2005).

[14] Ozaki, K., Tajika, E., Hong, P. K., Nakagawa, Y. & Reinhard, C. T. Effects of primitive photosynthesis on earth’s early climate system. *Nat. Geosci.* **11**, 55–59 (2018).

[15] Israël, G. et al. Complex organic matter in titan’s atmospheric aerosols from in situ pyrolysis and analysis. *Nature* **438**, 796–799 (2005). 16319825 10.1038/nature04349

[16] Arney, G. et al. The pale orange dot: the spectrum and habitability of hazy archean Earth. *Astrobiology* **16**, 873–899 (2016). 27792417 10.1089/ast.2015.1422 PMC5148108

[17] Haqq-Misra, J. D., Domagal-Goldman, S. D., Kasting, P. J. & Kasting, J. F. A revised, hazy methane greenhouse for the archean Earth. *Astrobiology* **8**, 1127–1137 (2008). 19093801 10.1089/ast.2007.0197

[18] Sagan, C. & Chyba, C. The early faint sun paradox: organic shielding of ultraviolet-labile greenhouse gases. *Science* **276**, 1217–1221 (1997). 11536805 10.1126/science.276.5316.1217

[19] Zerkle, A. L., Claire, M. W., Domagal-Goldman, S. D., Farquhar, J. & Poulton, S. W. A bistable organic-rich atmosphere on the Neoarchaean Earth. *Nat. Geosci.* **5**, 359–363 (2012).

[20] Izon, G. et al. Multiple oscillations in Neoarchaean atmospheric chemistry. *Earth Planet. Sci. Lett.* **431**, 264–273 (2015).

[21] Thomazo, C., Nisbet, E., Grassineau, N., Peters, M. & Strauss, H. Multiple sulfur and carbon isotope composition of sediments from the Belingwe greenstone belt (Zimbabwe): A biogenic methane regulation on mass independent fractionation of sulfur during the neoarchean?? *Geochim. Cosmochim. Acta*. **121**, 120–138 (2013).

[22] Flannery, D. et al. Spatially-resolved isotopic study of carbon trapped in ∼3.43 Ga Strelley pool formation stromatolites. *Geochim Cosmochim. Acta* **223**, (2017).

[23] Trainer, M. G. et al. Organic haze on Titan and the early Earth. *Proc. Natl. Acad. Sci. U. S. A.* **103**, 18035–18042 (2006). 10.1073/pnas.0608561103 PMC1838702 17101962

[24] Fleury, B. et al. Influence of CO on Titan atmospheric reactivity. *Icarus* **238**, 221–229 (2014).

[25] Atkinson, R. & Arey, J. Atmospheric degradation of volatile organic compounds. *Chem. Rev.* **103**, 4605–4638 (2003). 14664626 10.1021/cr0206420

[26] Rudich, Y., Donahue, N. M. & Mentel, T. F. Aging of organic aerosol: bridging the gap between laboratory and field studies. *Annu. Rev. Phys. Chem.* **58**, 321–352 (2007). 17090227 10.1146/annurev.physchem.58.032806.104432

[27] Sprengnether, M., Demerjian, K. L., Donahue, N. M. & Anderson, J. G. Product analysis of the OH oxidation of isoprene and 1,3-butadiene in the presence of NO. *J. Geophys. Res. Atmos.* **107**, ACH 8-1-ACH 8–13 (2002).

[28] Volkamer, R. et al. OH-initiated oxidation of benzene. *Phys. Chem. Chem. Phys.* **4**, 1598–1610 (2002).

[29] Brassé, C., Buch, A., Coll & Raulin, F. Low-Temperature alkaline pH hydrolysis of Oxygen-Free Titan tholins: carbonates’ impact. *Astrobiology* **17**, 8–26 (2017). 28103106 10.1089/ast.2016.1524

[30] Neish, C. D., Somogyi, Á. & Smith, M. A. Titan’s primordial soup: formation of amino acids via Low-Temperature hydrolysis of Tholins. *Astrobiology* **10**, 337–347 (2010). 20446873 10.1089/ast.2009.0402

[31] Khare, B. N. et al. Amino acids derived from Titan Tholins. *Icarus* **68**, 176–184 (1986). 11542046 10.1016/0019-1035(86)90080-1

[32] Derenne, S. et al. New insights into the structure and chemistry of titan’s Tholins via 13 C and 15 N solid state nuclear magnetic resonance spectroscopy. *Icarus* **221**, 844–853 (2012).

[33] Gautier, T. et al. Development of HPLC-Orbitrap method for identification of N-bearing molecules in complex organic material relevant to planetary environments. *Icarus* **275**, 259–266 (2016).

[34] Morisson, M., Szopa, C., Carrasco, N., Buch, A. & Gautier, T. Titan’s organic aerosols: molecular composition and structure of laboratory analogues inferred from pyrolysis gas chromatography mass spectrometry analysis. *Icarus* **277**, 442–454 (2016).

[35] Quirico, E. et al. New experimental constraints on the composition and structure of Tholins. *Icarus* **198**, 218–231 (2008).

[36] Maillard, J. et al. Humid evolution of haze in the atmosphere of Super-Earths in the habitable zone. *Astrobiology* **23**, 723–732 (2023). 37229532 10.1089/ast.2022.0021

[37] Poch, O., Coll, P., Buch, A., Ramírez, S. I. & Raulin, F. Production yields of organics of Astrobiological interest from H2O–NH3 hydrolysis of titan’s Tholins. *Planet. Space Sci.* **61**, 114–123 (2012).

[38] Jaziri, A. Y., Charnay, B., Selsis, F., Leconte, J. & Lefèvre, F. Dynamics of the great oxidation event from a 3D photochemical–climate model. *Clim. Past*. **18**, 2421–2447 (2022).

[39] Velivetskaya, T. A., Ignatiev, A. V., Vysotskiy, S. V. & Aseeva, A. V. Ratios of sulfur isotopes (32S, 33S, 34S, and 36S) in archean rocks of karelia: evidence of microbial life and the anoxic atmosphere. *Russ Geol. Geophys.* **65**, 689–698 (2024).

[40] Archer, C. & Vance, D. Coupled Fe and S isotope evidence for archean microbial Fe(III) and sulfate reduction. *Geology* **34**, 153–156 (2006).

[41] Craddock, P. R. & Dauphas, N. Iron and carbon isotope evidence for microbial iron respiration throughout the archean. *Earth Planet. Sci. Lett.* **303**, 121–132 (2011).

[42] Schopf, J. W. The fossil record of cyanobacteria. in Ecology of Cyanobacteria II: their Diversity in Space and time (ed Whitton, B. A.) 15–36 (Springer Netherlands, Dordrecht, doi:10.1007/978-94-007-3855-3_2. (2012).

[43] de Bueno, C. P., Wu, D. & Tringe, S. G. Methyl-Based methanogenesis: an ecological and genomic review. *Microbiol. Mol. Biol. Rev. MMBR*. **87**, e00024–e00022 (2013). 10.1128/mmbr.00024-22 PMC10029344 36692297

[44] Blaut, M. Metabolism of methanogens. *Antonie Van Leeuwenhoek*. **66**, 187–208 (1994). 7747931 10.1007/BF00871639

[45] Schauder, R. & Kröger, A. Bacterial sulphur respiration. *Arch. Microbiol.* **159**, 491–497 (1993).

[46] Ebrahiminezhad, A., Manafi, Z., Berenjian, A., Kianpour, S. & Ghasemi, Y. Iron-Reducing bacteria and iron nanostructures. *J. Adv. Med. Sci. Appl. Technol.* **3**, 9 (2017).

[47] Bonch-Osmolovskaya, E. A., Sokolova, T. G., Kostrikina, N. A. & Zavarzin, G. A. Desulfurella acetivorans gen. Nov. And sp. Nov. —a new thermophilic sulfur-reducing Eubacterium. *Arch. Microbiol.* **153**, 151–155 (1990).

[48] Straub, K. L. & Buchholz-Cleven, B. E. Geobacter Bremensis sp. Nov. And geobacter pelophilus sp. Nov., two dissimilatory ferric-iron-reducing bacteria. *Int. J. Syst. Evol. Microbiol.* **51**, 1805–1808 (2001). 11594612 10.1099/00207713-51-5-1805

[49] Veglió, F., Passariello, B., Barbaro, M., Plescia, P. & Marabini, A. M. Drum leaching tests in iron removal from quartz using oxalic and sulphuric acids. *Int. J. Min. Process.* **54**, 183–200 (1998).

[50] Veaudor, T., Cassier-Chauvat, C. & Chauvat, F. Genomics of Urea transport and catabolism in cyanobacteria: biotechnological implications. *Front. Microbiol.* **10**, 2052 (2019). 31551986 10.3389/fmicb.2019.02052 PMC6737895

[51] Solomon, C., Collier, J., Berg, G. & Glibert, P. Role of Urea in microbial metabolism in aquatic systems: a biochemical and molecular review. *Aquat. Microb. Ecol.* **59**, 67–88 (2010).

[52] Grettenberger, C. L. et al. A phylogenetically novel Cyanobacterium most closely related to Gloeobacter. *ISME J.* **14**, 2142–2152 (2020). 32424249 10.1038/s41396-020-0668-5 PMC7368068

[53] Lahav, N., White, D. & Chang, S. Peptide formation in the prebiotic era: thermal condensation of Glycine in fluctuating clay environments. *Science* **201**, 67–69 (1978). 663639 10.1126/science.663639

[54] Wolf, E. T. & Toon, O. B. Fractal organic hazes provided an ultraviolet shield for early Earth. *Science* **328**, 1266–1268 (2010). 20522772 10.1126/science.1183260

[55] Canfield, D. E., Rosing, M. T. & Bjerrum, C. Early anaerobic metabolisms. *Philos. Trans. R Soc. B Biol. Sci.* **361**, 1819–1836 (2006). 10.1098/rstb.2006.1906 PMC1664682 17008221

[56] Szopa, C., Cernogora, G., Boufendi, L., Correia, J. J. & Coll, P. PAMPRE: A dusty plasma experiment for titan’s Tholins production and study. *Planet. Space Sci.* **54**, 394–404 (2006).

