# Supplementary material for: Transient oxidation of hazes a source of nutrients during the great oxidation event
Source: Sci Rep. 2025 Aug 11;15:29284. doi: 10.1038/s41598-025-13441-1 (PMC12336318; doi:10.1038/s41598-025-13441-1)
Supplement: Supplementary file 1 — Supplementary Material 1 [file 41598_2025_13441_MOESM1_ESM.docx]

**Supplementary materials**


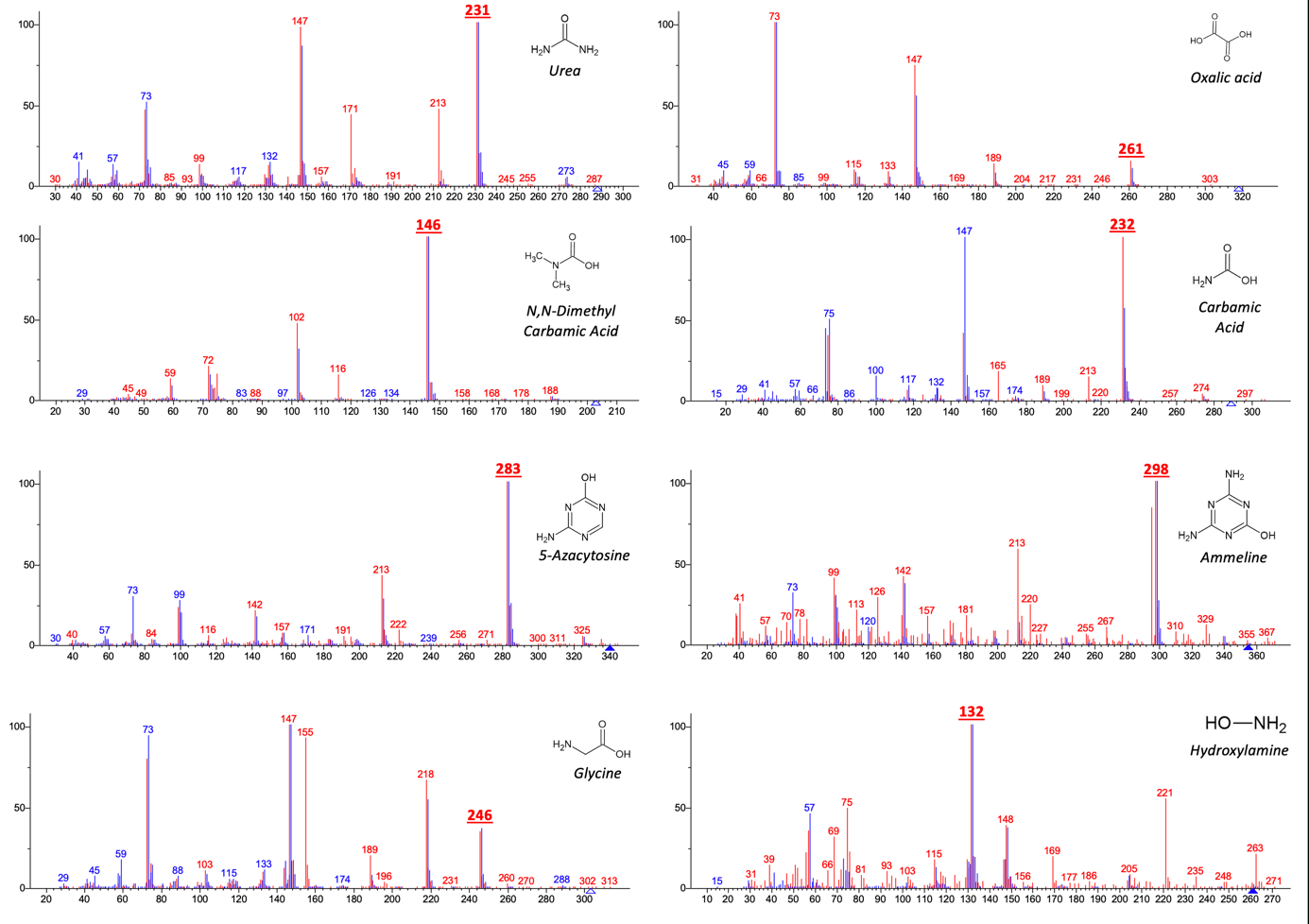


Figure S-1 : Comparison between experimental mass spectra of the sample from the first replicate in red and the corresponding standard molecules spectra in blue. The mass fragments used to obtain the signals Figure 1 are underlined and bolded. The blue triangle indicates the molecular peak of the species. All the molecules are derivatized two times (with two Tert-ButylDiMethylSilyl t-BDMS groups instead of labile hydrogens) except for N,N-Dimethyl Carbamic acid which is derivatized only one time. It explains the high molecular mass observed for the species due to the presence of this heavy TBDMS group. The supplementary peaks observed in the sample spectra in red are for the big majority of them due to noise contribution. Only the peaks at m/z=171 and 213 for urea, and m/z=165 and 213 for carbamic acid are not in the standard reference spectra and meaningfull. Such peaks are attributed to co-eluted species.

**
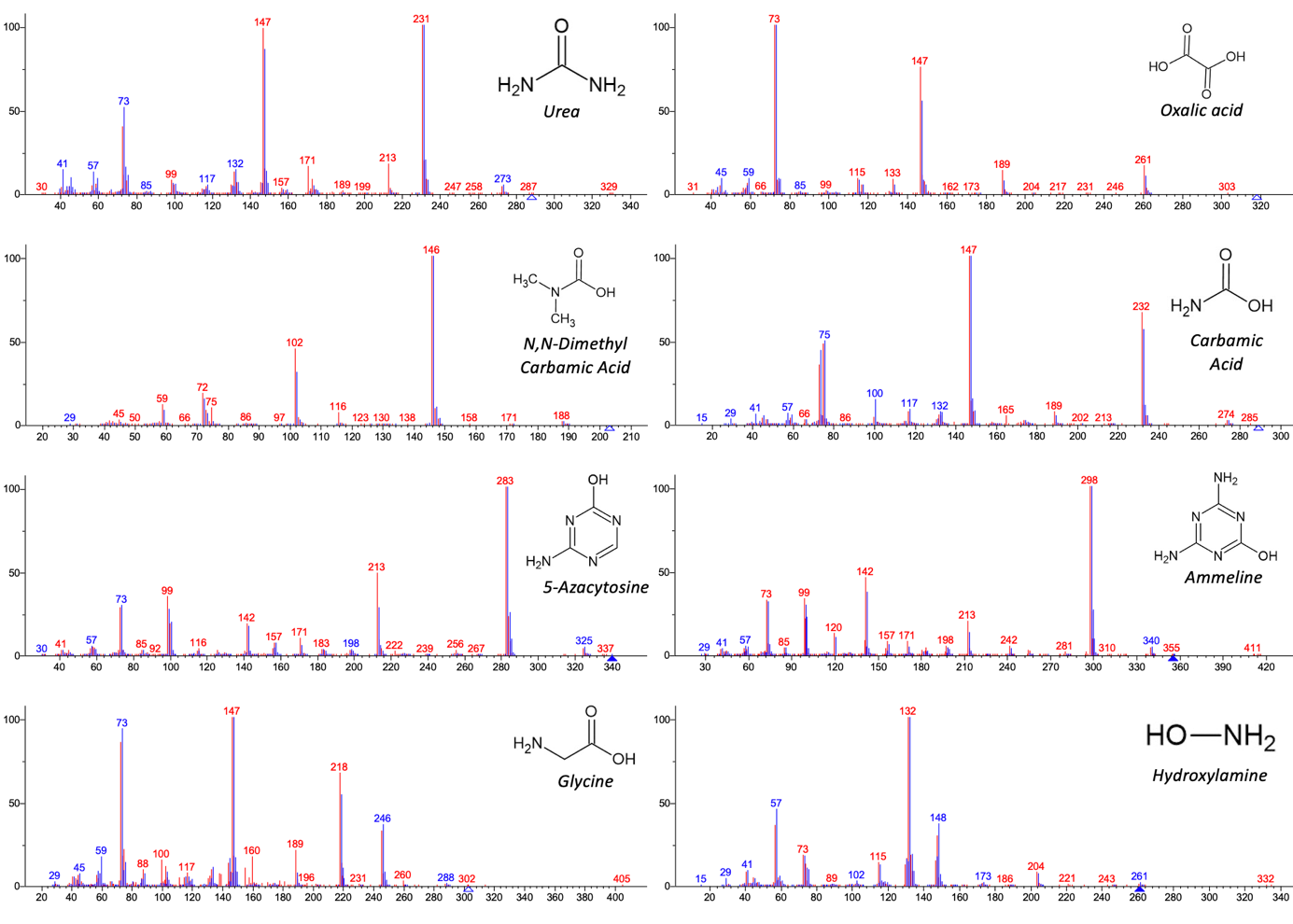
**

Figure S-2: As done in Figure 2, comparison between experimental mass spectra of the sample in red and the corresponding standard molecules spectra in blue. For this figure, aerosols have been exposed voluntary for ten days to an ambient atmosphere under more oxidizing conditions than in Figure S-1. In this way, oxidation processes are accelerated to produce more oxygenated organics and improve their signatures. All the specific fragments of the eight molecules displayed Figure S- 1 are observed at the same retention time which finally confirm their detection. In these conditions, the signal over noise ratio is significatively improved which allow the disappearance of numerous peaks observed in the mass spectra Figure S-1. It confirms that the majority of the supplementary peaks observed Figure S-1 are due to noise contribution.


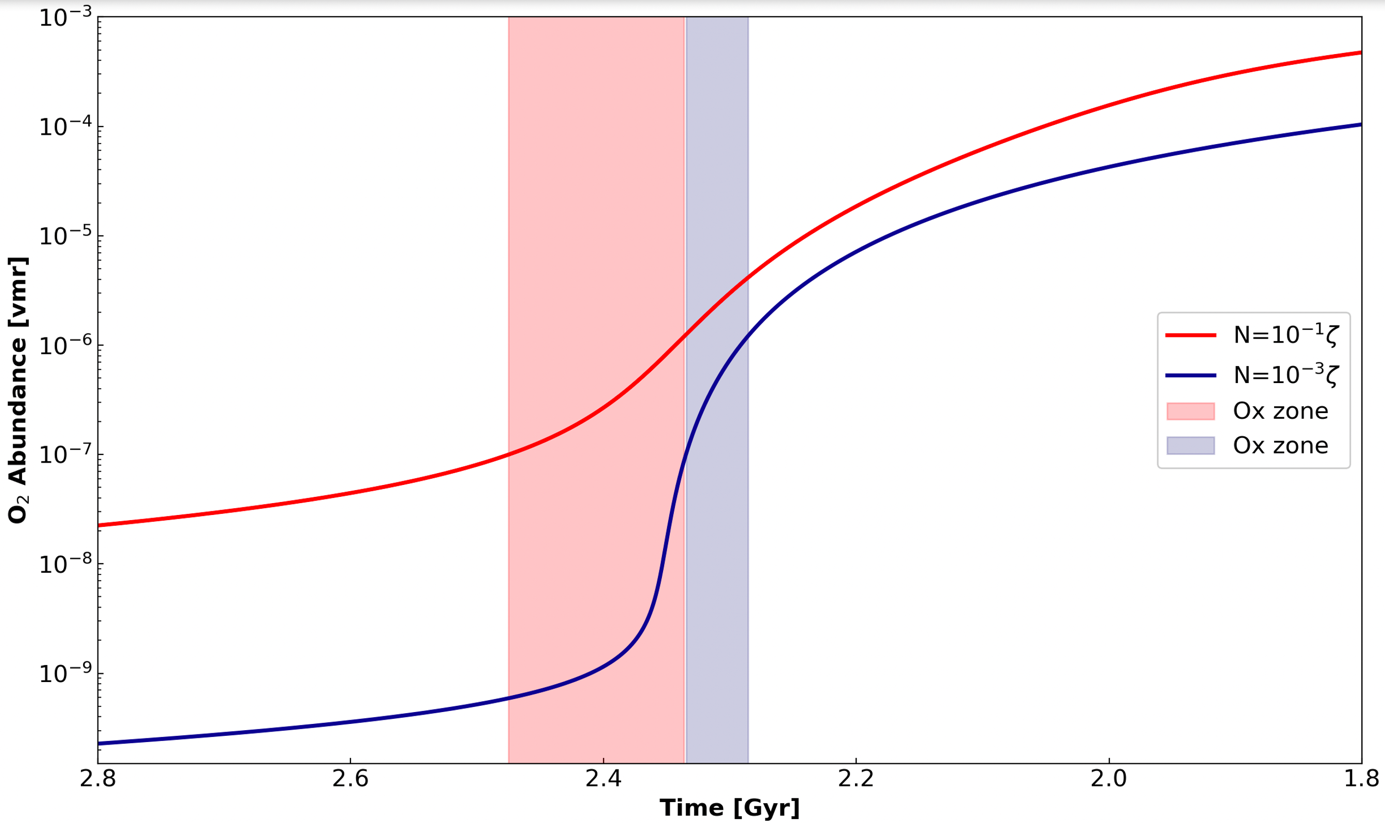


Figure S-3: O_2_ temporal evolution profiles during the GOE assuming different net primary oxygen productivities ($N={10}^{-1}\zeta$, $N={10}^{-3}\zeta$ with $\zeta=3.75\times{10}^{14}$mol O_2_.yr^—1^) and their corresponding aerosol oxidation windows. In the case of a less productive system in blue, the aerosol oxidation period is delayed and shortened. Lower productivity implies a more reduced atmosphere during the Archean in which the oxidation threshold of 0.1 ppm is reached later (at 2.34 Gyrs in blue versus 2.47 in red) and aerosols can subsist longer (up to 2.28 Gyrs in blue versus 2.33 in red). Moreover, the steeper slope in the O_2_ profile at $N={10}^{-3}\zeta$ explains the shorter oxidation zone observed in this case. Methane is consumed more rapidly once the oxidation threshold is reached, so aerosols disappear more quickly.
